# Purification and Properties of an Insecticidal Metalloprotease Produced by *Photorhabdus luminescens* Strain 0805-P5G, the Entomopathogenic Nematode Symbiont

**DOI:** 10.3390/ijms14010308

**Published:** 2012-12-21

**Authors:** Yu-Tzu Chang, Chienyan Hsieh, Li-Ching Wu, Hebron C. Chang, Suey-Sheng Kao, Menghsiao Meng, Feng-Chia Hsieh

**Affiliations:** 1 Institute of Biotechnology and Bioinformatics, Asia University, Wufeng, Taichung 413, Taiwan; E-Mails: yyya.tw@yahoo.com.tw (Y.T.C.); hebronchang2005@yahoo.com (H.C.C.); 2 Department of Biotechnology, National Kaohsiung Normal University, Kaohsiung 824, Taiwan; E-Mail: mch@nknucc.nknu.edu.tw; 3 Biopesticides Division, Taiwan Agricultural Chemicals and Toxic Substances Research Institute, Council of Agriculture, Wufeng, Taichung 413, Taiwan; E-Mails: 456457@yahoo.com.tw (L.-C.W.); sskao@tactri.gov.tw (S.-S.K.); 4 Graduate Institute of Biotechnology, National Chung Hsing University, Taichung 402, Taiwan

**Keywords:** protease, *Photorhabdus luminescens*, *Plutella xylostella*, insecticidal activity

## Abstract

A total of 13 *Photorhabdus luminescens* strains were screened for proteolytic activity. The *P. luminescens* strain 0805-P5G had the highest activity on both skim milk and gelatin plates. The protease was purified to electrophoretical homogeneity by using a two-step column chromatographic procedure. It had a molecular weight of 51.8 kDa, as determined by MALDI-TOF mass spectrometry. The optimum pH, temperature, as well as pH and thermal stabilities were 8, 60 °C, 5–10, and 14–60 °C, respectively. It was completely inhibited by EDTA and 1,10-phenanthroline. Bioassay of the purified protease against *Galleria mellonella* by injection showed high insecticidal activity. The protease also showed high oral toxicity to the diamondback moth (*Plutella xylostella*) of a Taiwan field-collected strain, but low toxicity to an American strain. To our knowledge, this is the first report to demonstrate that the purified protease of *P. luminescens* has direct toxicity to *P. xylostella* and biopesticide potentiality.

## 1. Introduction

*Photorhabdus luminescens* is an insect-pathogenic gram-negative, rod-shaped bacterium that was isolated from light-emitting insects that had been infected by entomogenous nematodes of the family *Heterorhabditidae*. The entomopathogenic *Photorhabdus* lives in symbiosis with nematodes that invade insects. Following entry into the insect, the bacteria are released from the nematode gut into the insect hemocoel. *Photorhabdus* secretes toxins and other potentially virulent factors, including proteases and lipases [1,2], which kill the host and also convert host tissues into food (“bioconversion”) for both the replicating bacteria and nematodes [3]. Bowen *et al.* [4,5] reported that injected protease was not directly toxic to third-instar *Manduca sexta* or fifth-instar *Galleria mellonella* larvae. However, some researchers [6–8] suggested that toxicity is correlated with the protease activity. So far, conclusive evidence supporting this assumption has not been presented. To further clarify the likely role of the secreted protease, here the purification, characterization and insecticidal activity of an alkaline protease extracellularly produced by our newly isolated *P. luminescens* strain 0805-P5G was studied. Although the tobacco hornworm *M. sexta* has been used extensively as a model host for insect physiology, it’s not always the best choice. *Plutella xylostella* is a possible replacement candidate, for it is an important and cosmopolitan pest of cruciferous crops with a global distribution. This is the first report that purified protease of *P. luminescens* is in fact toxic to two different insect models, *G. mellonella* and *P. xylostella*, via injection or ingestion and is likely to aid the understanding of its pathogenic role and use as a resource for GMO (Genetically Modified Organism).

## 2. Results and Discussion

### 2.1. Strain Isolation and Identification

**Figure 1:**
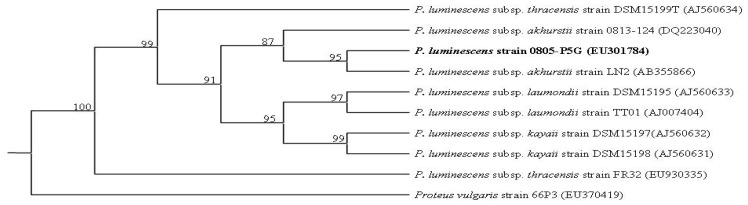
Strict consensus of most parsimonious tree inferred by maximum parsimony analyses of *Photorhabdus* spp. 16S rDNA sequences data set. GenBank accession numbers are in parentheses after the strain names. Bootstrap values (percentages of 1000 replicates) are shown at nodes. The *Photorhabdus* sp. used in our study is in bold font.

A bacterial strain was selected from others, isolated from infective juveniles (IJs) of *Heterorhabditis brevicaudis* YS for further work, because it had the highest protease activity based on the clear zone formation around the colonies grown on skim milk agar and on gelatin agar plates. The result of Biolog identification system only supported the identification of the bacterial strain as a *Photorhabdus* sp. (data not shown). The 16S rDNA sequence of the strain 0805-P5G was deposited in the GeneBank database under accession number EU301784. After analyzing phylogenetic trees, the strain 0805-P5G was most related to *P. luminescens* subsp. *akhurstii* strain LN2, it was proposed be within the *P. luminescens* subsp. *akhurstii* (Figure 1). We found that the *P. luminescens* strain 0805-P5G secreted protease from the early logarithmic phase of growth in a variety of media, such as NB, LB and PP3T. The maximum proteolytic activity was obtained from the NB culture medium at eight days post-inoculation with time-course data (data not shown).

### 2.2. Purification of Protease from *P. luminescens*

**Figure 2:**
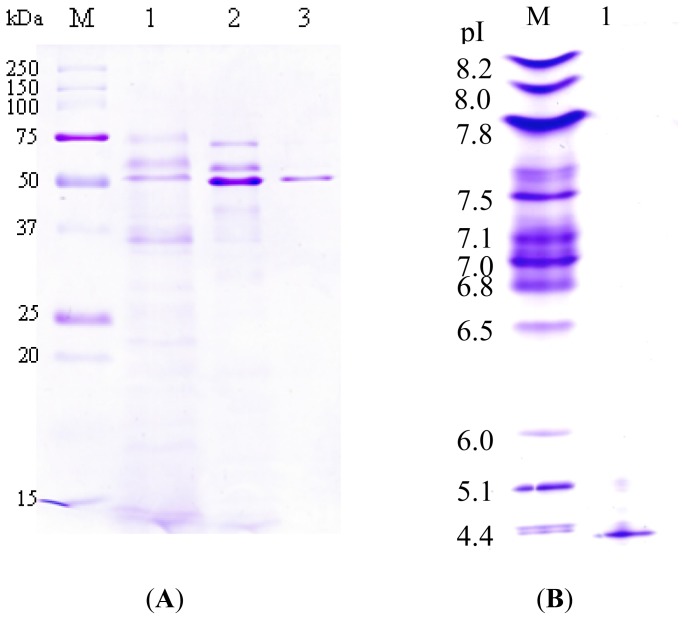
Analysis of the purified protease by (**A**) SDS-PAGE and (**B**) IEF. (**A**) M, molecular weight marker proteins; Lane 1, supernatant of *P. luminescens* strain 0805-P5G culture; Lane 2, protease fraction from DEAE Sepharose column; Lane 3, protease fraction from Q Sepharose column. (**B**) M, isoelectric point marker proteins; Lane 1, purified protease from Q Sepharose column.

The homogeneity of the protease from *P. luminescens* 0805-P5G was greatly increased after the purification process by FPLC using DEAE column and Q Sepharose column. The final specific activity, fold of purification and recovery is 85.7 U/mg, 10.6 and 8.2%, respectively. The isoelectric point of the protease is 4.2 (Figure 2B).

### 2.3. SDS-PAGE and Zymograms

**Figure 3:**
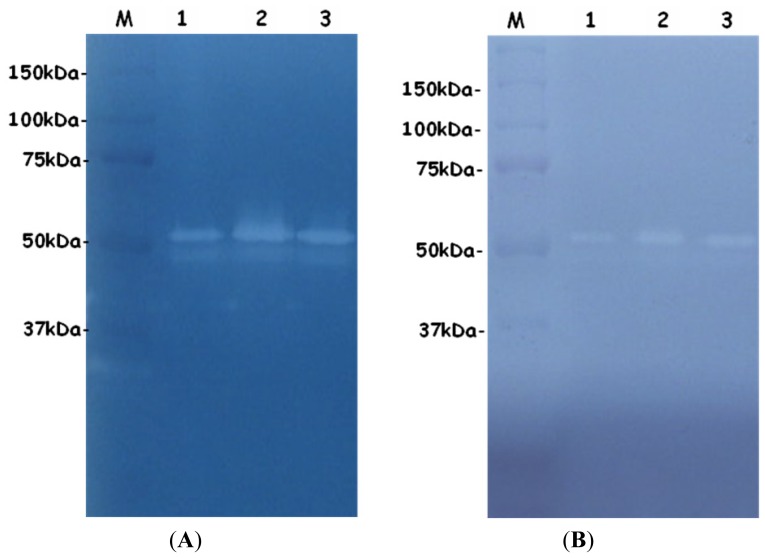
Enzyme activity staining of protease from *Photorhabdus luminescens* strain 0805-P5G at different purification steps by zymograms. (**A**) Gelatin zymogram showing region of clearing associated with protease activities. M: molar mass marker (Bio-Rad); Lane1: Supernatant of *Photorhabdus luminescens* strain 0805-P5G culture; Lane2: protease fraction from DEAE Sepharose column; Lane3: protease fraction from Q Sepharose column. (**B**) Casein zymogram (lanes as in panel A).

The authenticity of the protease was confirmed by SDS-PAGE and zymograms. The purified protease showed a single band of 51 kDa in SDS-PAGE (Figure 2A) and exhibited proteolytic activity at the corresponding bands in the zymogram with gelatin and casein, respectively (Figure 3).

### 2.4. *N*-terminal Amino Acid Sequence and Mass Spectrometry

The *N*-terminal amino acid sequencing of the purified protease showed the first 10 amino acid residues, DKDVSGSEKA, which is homologous to alkaline metalloprotease from *P. luminescens* subsp. *akhurstii* W14 and *P. luminescens* subsp. *laumondii* TT01 [9]. Furthermore, the partial peptide sequence of the protease was identified by LC MS/MS (data not shown). The molecular mass of the purified protease was determined by MALDI-TOF mass spectrometry to be 51.8 kDa, consistent with the results of SDS-PAGE (Figure 2A) and zymograms (Figure 3).

### 2.5. Effect of pH on Enzyme Activity and Stability

**Figure 4:**
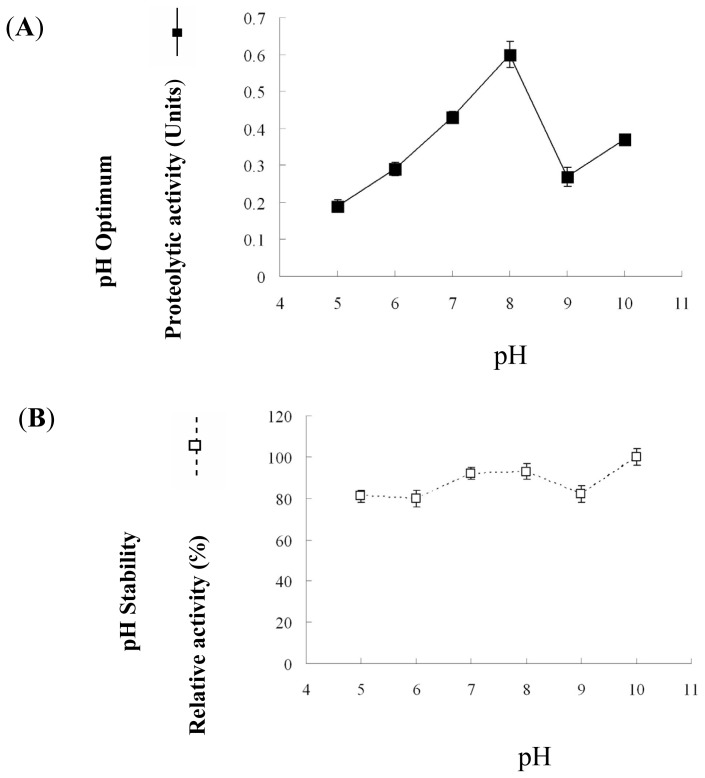
Optimum pH range of activity (**A**) and stability (**B**) of the *P. luminescens* protease. The protease was incubated in 200 mM Tris-HCl buffer at 60 °C for 30 min. For the buffers used and the conditions of the measurement, see the Experimental Section.

The protease of *P. luminescens* strain 0805-P5G exhibited highest activity at pH 8 (Figure 4). It remained stable in a wide pH range (5–10) and retained more than 80% of its original activity after incubation for 30 min.

### 2.6. Effect of Temperature on Enzyme Activity and Stability

**Figure 5:**
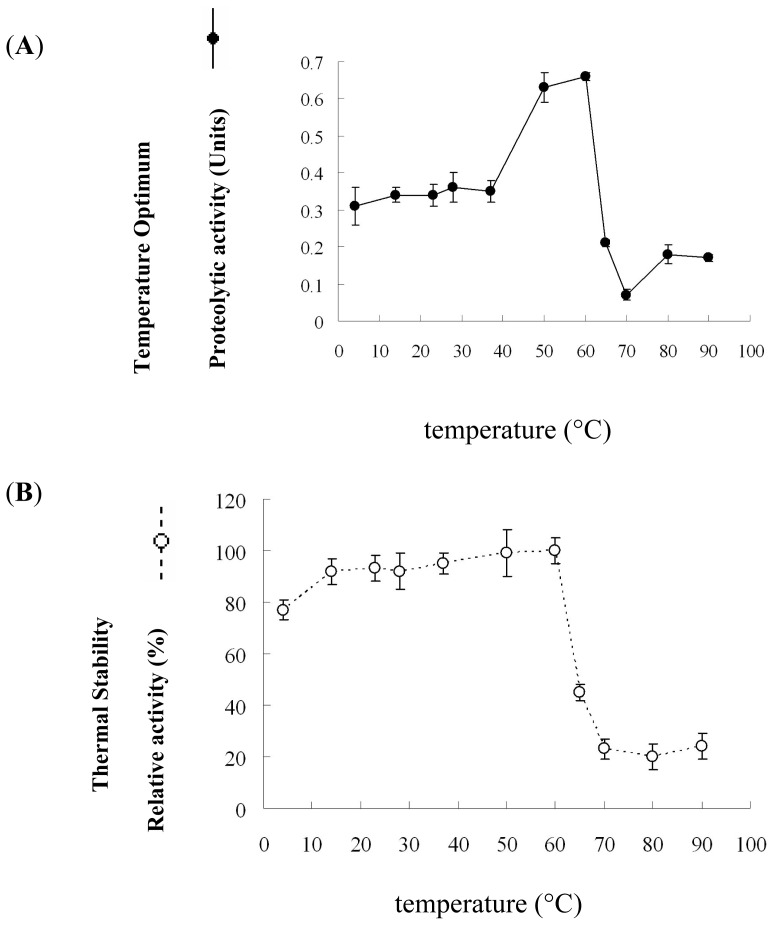
Optimum temperature range of activity (**A**) and stability (**B**) of the *P. luminescens* protease. For the determination of short-term temperature stability assay, the protease was incubated in 200 mM Tris-HCl buffer pH 8 for 30 min. For the buffers used and the conditions of the measurement, see the Experimental Section.

Results showed that the protease had an optimum of 60 °C, with 35% activity retained at 90 °C (Figure 5). In addition, thermal stability results also showed that this enzyme had nearly 100% residual activity in the temperature range of 14–60 °C for 30 min (Figure 5). The long thermal stability showed that the protease is stable, with less than 50% loss in enzyme activity observed upon incubation, even after 10 days at 60 °C (data not shown).

### 2.7. Effect of Protease Inhibitors on Enzyme Activity

**Figure 6:**
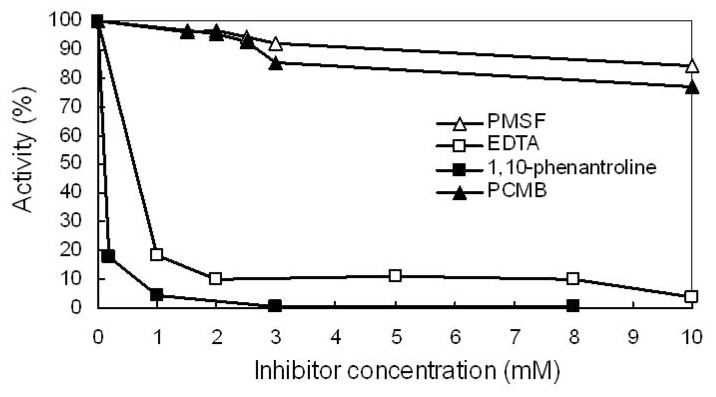
Inhibition of protease by protease inhibitors. The inhibited activity of the purified protease is expressed as a percentage of the uninhibited activity. Open triangles, PMSF, a serine protease inhibitor; filled triangles, PCMB, a thiol protease inhibitor; open squares, EDTA, a metalloprotease inhibitor; filled squares, 1,10-phenanthroline, a zinc metalloprotease inhibitor, but not specific for Zn. For the conditions of the measurement, see the Experimental Section.

Little inhibition was observed with PMSF and PCMB (Figure 6), which are inhibitors of serine and thiol proteases, respectively. However, significant levels of inhibition were observed following incubation with either EDTA (a general metalloprotease inhibitor) or 1,10-phenanthroline (a zinc metalloprotease inhibitor, but not specific for Zn). There was also a dose-dependent response to these two inhibitors at concentrations between 0.2 and 10 mM (Figure 6). These data are consistent with the supposition that the enzyme might be a Zn metalloprotease.

### 2.8. Insect Toxicity Assay

The bioassay of *P. luminescens* protease against *G. mellonella* by injection showed high insecticidal activity. It had the significant insecticidal rate of 100% at the concentration of 11.6 ng/μL. The insecticidal activity decreased clearly when the concentration was decreased (Table 1). The LC_50_ value against *G. mellonella* after injection is 3.8 ng/μL (2.1–4.7 ng/μL). The numbers in parentheses are the 95% fiducial limits. In another experiment to test the oral toxicity, both the field-collected and the American strains of *P. xylostella* were assayed, only the field-collected strain from Taiwan showed high oral toxicity (LC_50_ is 43 ng/μL). In contrast, low oral toxicity (LC_50_ is much more than 120 ng/μL) was shown against the American strain of *P. xylostella*. A general authentic protease (purified from *Bacillus polymyxa*) purchased from Sigma, with the same concentrations were used both orally and in injection to serve control to both experiments. The results showed that control treatments were non-toxic to insects. Another result of the verification experiment of the purified metalloprotease as the cause of insect toxicity (injected) showed the two controls, both at 3.8 ng/μL, one with 1,10-phenantroline treated then dialyzed enzyme significantly decreased mortality rate (3%), the other with only dialyzed enzyme preparation retained mortality rate (47%).

## 3. Experimental Section

### 3.1. Media, Growth Conditions and Protease Activity Assay

Thirteen local *Photorhabdus luminescens* strains were maintained on NBTA plates [(2.3% nutrient agar (Difco, Franklin Lakes, NJ, USA), 0.0025% bromothymol blue (Merck, Whitehouse Station, NJ, USA), 0.004% 2,3,5-triphenyltetrazolium (Sigma-Aldrich, St. Louis, MO, USA)] at 30 °C and subcultured weekly. 250-mL Erlenmeyer flasks with 50 mL of three different liquid culture media, namely, NB (nutrient broth, Difco, Franklin Lakes, NJ, USA), LB broth (Luria-Bertani Miller, Difco, Franklin Lakes, NJ, USA) and PP3T [(2% Proteose Peptone No. 3 (Difco, Franklin Lakes, NJ, USA) plus 0.5% Tween 60] were inoculated, respectively. The proteolytic activity during bacterial growth was monitored daily by a caseinolytic macroassay. The protease activity in the fractions obtained in the purification steps was tested by a microassay. The macroassay and the microassay of protease activity were both performed as described by Cabral *et al.* [1]. Aliquots (50 μL) of culture supernatant were obtained at different time intervals, combined with 80 μL of 2% azocasein (Sigma) in 50 mM Tris-HCl (pH 8.5) and incubated at 37 °C for 3 h. The undigested substrate was precipitated by adding 400 μL of 10% trichloroacetic acid to the reaction mixture, followed by incubation for 10 min at 4 °C, followed in turn by centrifugation at 10,000× *g* for 10 min. The supernatant was neutralized by the addition of 600 μL of 1 N NaOH, and the absorbance at 440 nm (A_440_) was measured. The protease activity in the fractions obtained in the purification steps was tested by a microassay for which 20-μL aliquots from each fraction were added to 40 μL of 3% azocasein in Tris, plus an additional 40 μL of 50 mM Tris-HCl (pH 8.5), followed by incubation at 37 °C for 30 min. The undigested substrate was precipitated by adding 75 μL of 10% trichloroacetic acid to the reaction mixture, followed by incubation for 10 min at 4 °C and centrifugation at 10,000× *g* for 10 min. Then, 100 μL of supernatant was transferred to a 96-well microtitration plate and neutralized by the addition of an equal volume of 1 N NaOH. The A_440_ was measured by using a microplate reader (Thermo Scientific Inc., Waltham, MA, USA). One unit of enzyme activity was defined as the amount of enzyme that yielded an absorbance change of 0.01.

### 3.2. Screening for Protease Activity

Proteases production by 13 local *P. luminescens* was detected on skim milk and gelatin agar plates, which were incubated at 30 °C for five days. Skim milk agar contained 1.5% skim milk powder (Difco, Franklin Lakes, NJ, USA), 1.3% nutrient broth (Scharlau) and 1.5% agar (Oxoid), while skim milk powder was replaced by gelatin in gelatin agar. Microorganisms with proteolytic activity were detected by the formation of clear zones around colonies.

### 3.3. Strain Identification

Symbiotic bacteria of nematode *Heterorhabditis brevicaudis* YS were observed by culturing on MacConkey’s agar and NBTA plates. Of them, a *P. luminescens* strain 0805-P5G was isolated. The 16S rDNA sequences and phylogenetic analyses were conducted as described in our previous study [10]. The 96-well Biolog GN2 microplate (Biolog Inc., Hayward, CA, USA) was also used to assay the oxidation of 95 carbon substrates as described in our previous study [6].

### 3.4. Purification of Protease from *P. luminescens*

A two-column strategy was developed for protease purification. All the purification steps were performed at 4 °C with AKTKprime (GE Healthcare Bio-Sciences, Pittsburgh, PA, USA). Following growth on NB media, 150 mL of eight-day culture was centrifuged at 30,000× *g* for 30 min at 4 °C. The supernatant was then applied to a HiTrap DEAE Sepharose Fast Flow column equilibrated with 50 mM K_2_PO_4_ (pH 8.6). The bound proteins were eluted in a linear gradient of 8 mL from 0 to 0.2 M KCl in 50 mM K_2_PO_4_ (pH 9.3), followed by a linear gradient of 10 mL from 0.2 to 0.25 M KCl and, finally, a linear gradient of 5 mL from 0.25 to 1.0 M KCl. The fractions with protease activity were then pooled, dialyzed against 50 mM Tris-HCl (pH 8.5) and loaded onto a HiTrap Q Sepharose XL column equilibrated with the same buffer. The bound proteins were eluted with 40 mL of NaCl in a linear gradient of 0 to 1.0 M in 50 mM Tris-HCl (pH 8.5). The protease activity in the fractions obtained in the purification steps was assayed by a microassay as described by Cabral *et al.* [1].

### 3.5. SDS-PAGE and Zymograms

Sodium dodecyl sulfate-polyacrylamide gel electrophoresis (SDS-PAGE) was performed in 12% polyacrylamide gel. *P. luminescens* protease has high affinity for substrates, such as casein and gelatin [5]; therefore, zymograms were performed as described by Schmidt *et al.* [11] and Cabral *et al.* [1], with minor modifications. Polyacrylamide gels (12%) were copolymerized with 0.05% gelatin or casein. The samples were applied in nonreducing Laemmli buffer without boiling and run at 100 V. The gels were washed twice in 2.5% (*w*/*v*) Triton X-100 solution and then incubated in 50 mM Tris-HCl (pH 7.5) with 5 mM CaCl_2_ and 200 mM NaCl for 2 h at 30 °C [5]. The gels were stained with 0.1% amido black in methanol-acetic acid-water (40:10:50, *v*/*v*/*v*) and later washed until the zones of substrate hydrolysis were visible. Protease requires bound calcium to stabilize its structure [5], and we therefore added 5 mM CaCl_2_ to the development buffer of zymography.

### 3.6. Isoelectric Focusing (IEF)

Polyacrylamide isoelectric focusing (IEF) was performed in a Mini-PROTEAN 3 gel system (Bio-Rad) using ampholytes of the pH range 3 to 10. Approximately 3–5 μg of the purified protein along with standards (pI 4.45–9.6) were applied to the ready gel (Bio-Rad) according to the manufacturer’s instructions. The IEF gel was stained with Coomassie brilliant blue.

### 3.7. *N*-terminal Amino Acid Sequence and Mass Spectrometry

The *N*-terminal amino acids of purified protease were sequenced by automated Edman degradation. The exact molecular mass of the purified protease was determined by matrix-assisted laser desorption ionization-time-of-flight (MALDI-TOF) mass spectrometry. Both techniques were carried out by the Mission Biotech Company (Taipei, Taiwan).

### 3.8. Effect of pH on Enzyme Activity and Stability

Twenty μl of the purified proteases (116 ng/μL) activity was determined by varying the pH of the assay reaction mixture using the following buffers: 200 mM glycine (pH 3), 200 mM sodium acetate (pH 4–5), 200 mM sodium phosphate (pH 6), 200 mM Tris-HCl (pH 7–8) and 200 mM Tris-glycine (pH 9–10). To determine the stability of the protease, it was preincubated in different buffers (pH 3–10) for 30 min. The maximum activity was defined as 100%. The relative activity (% of maximum) was calculated. The averages from three experiments are shown. The proteolytic activity was accessed by using the azocasein microassay described as Section 3.1. Twenty microliter of the purified proteases (116 ng/μL) was added to 40 μL of 3% azocasein in Tris plus an additional 40 μL of 50 mM Tris-HCl (pH 8.5), followed by incubation at 37 °C for 30 min. The undigested substrate was precipitated by adding 75 μL of 10% trichloroacetic acid to the reaction mixture, followed by incubation for 10 min at 4 °C and centrifugation at 10,000× *g* for 10 min. Then, 100 μL of supernatant was transferred to a 96-well microtitration plate and neutralized by the addition of an equal volume of 1 N NaOH. The A_440_ was measured by using a microplate reader (Thermo Scientific Inc., Waltham, MA, USA).

### 3.9. Effect of Temperature on Enzyme Activity and Stability

The activity was evaluated by measuring 20 μL of the purified proteases (116 ng/μL) activity at 4, 14, 23, 28, 37, 50, 60, 65, 70, 80 and 90 °C in 50 mM Tris-HCl (pH 8) for 30 min. The effect of temperature on protease stability was determined by measuring the residual activity after 30 min of pre-incubation at different temperature. The maximum activity was defined as 100%. The relative activity (% of maximum) was calculated. The averages from three experiments are shown. The long-term stability of the enzyme was determined by measuring the residual activity at one-day intervals for 10 days of pre-incubation at 60 °C. The proteolytic activity was accessed by using the azocasein microassay described as Section 3.1. Twenty microliter of the purified proteases (116 ng/μL) was added to 40 μL of 3% azocasein in Tris, plus an additional 40 μL of 50 mM Tris-HCl (pH 8.5), followed by incubation at 37 °C for 30 min. The undigested substrate was precipitated by adding 75 μL of 10% trichloroacetic acid to the reaction mixture, followed by incubation for 10 min at 4°C and centrifugation at 10,000× *g* for 10 min. Then, 100 μL of supernatant was transferred to a 96-well microtitration plate and neutralized by the addition of an equal volume of 1 N NaOH. The A_440_ was measured by using a microplate reader (Thermo Scientific Inc., Waltham, MA, USA).

### 3.10. Effect of Protease Inhibitors on Enzyme Activity

Twenty microliter of the purified proteases (116 ng/μL) were incubated for 30 min at room temperature in the presence of phenylmethanesulfonyl fluoride (PMSF) (1.5, 2, 2.5, 3, and 10 mM), *p*-chloromercuribenzoate (PCMB) (1.5, 2, 2.5, 3, and 10 mM), ethylenediaminetetraacetic acid (EDTA) (1, 2, 5, 8, and 10 mM) and 1,10-phenantroline (0.2, 1, 3, and 8 mM). The activity assayed in the absence of protease inhibitors was defined as the reference control. The remaining proteolytic activity was accessed by using the azocasein microassay described as Section 3.1. Twenty microliters of the purified proteases (116 ng/μL) was added to 40 μL of 3% azocasein in Tris, plus an additional 40 μL of 50 mM Tris-HCl (pH 8.5), followed by incubation at 37 °C for 30 min. The undigested substrate was precipitated by adding 75 μL of 10% trichloroacetic acid to the reaction mixture, followed by incubation for 10 min at 4 °C and centrifugation at 10,000× *g* for 10 min. Then, 100 μL of supernatant was transferred to a 96-well microtitration plate and neutralized by the addition of an equal volume of 1 N NaOH. The A_440_ was measured by using a microplate reader (Thermo Scientific Inc., Waltham, MA, USA). The averages from three experiments are shown.

### 3.11. Insect Toxicity Assay

For the *Galleria* haemolymph injection bioassay, aliquot 10 μL of each sample (11.6 ng/μL, 9.5 ng/μL, 7.3 ng/μL, 6.5 ng/μL, 5.8 ng/μL, 4.8 ng/μL and 3.7 ng/μL; the purified proteases were diluted with 50 mM Tris-HCl containing 250 mM NaCl.), which had been filter sterilized, was injected into seventh-instar greater wax moth (*G. mellonella*) aseptically using a 10 μL syringe (Hamilton). The oral bioassay was performed also with two strains (a field-collected strain and an American strain) of laboratory-reared diamondback moth (*P. xylostella*) under constant conditions (temperature 25 °C, relative humidity 60%–70%, under 15/9 h light/dark photoperiod). The field strain was collected locally, and the American strain was kindly provided by Syngenta (Wilmington, DE, USA) Ltd. *P. xylostella* larvae reared on the surface of the artificial diet that contain more than 10 ingredients [12] and can be effectively assayed using the surface contamination method. Proteases to be assayed orally were mixed with an appropriate artificial diet and fed to third-instar larvae at 25 °C. A general authentic protease (purified from *Bacillus polymyxa*) purchased from Sigma with the same concentrations were used both orally and by injection to serve control treatments (non-insect toxic) to both experiments to justify insect specificity of the isolated protease. The mortality rate for each concentration for 30 insect larvae was recorded after incubation for three days at 25 °C. The concentration-mortality regressions were then evaluated. At least five concentrations resulting in mortalities >10% and <90% were desirable. The data for 50% lethal concentrations (LC_50_), which were estimated using a Probit analysis program [13], were the averages from three experiments. A further verification experiment of the purified metalloprotease as the cause of insect toxicity (oral and injected) was performed. Two controls, one with 1,10-phenantroline treated then dialyzed enzyme, the other with only dialyzed enzyme preparation at the same concentration were assayed.

## 4. Discussion

Previous reports of culture supernatants of *Photorhabdus* spp. produced conflicting results on the role of extracellular protease in insect toxicity. Zinc metalloproteases are common in pathogenic bacteria and are often attributed roles in virulence [6,7,14–17]. Metalloprotease is also produced by several species of insect pathogens, including *Pseudomonas aeruginosa*, *Proteus mirabilis* and *Serratia marcescens. Photorhabdus* delivers protease into the insect hemocoel is by means of an insect pathogen, such as entomopathogenic nematodes (just like “injection”). These metalloproteases are thought to induce death in infected insects by specifically hydrolyzing hemolymph proteins and causing damage to the hemocytes [7]. Yamanaka *et al.* [8] reported low injectable insecticidal activity for *Spodoptera litura* (Lepidoptera, Noctuidae) larvae in culture broths of only one of three *Photorhabdus* strains. But all three extracts contained high levels of protease activity, leading the authors to conclude that proteases are not involved in virulence. Schmidt *et al.* [11] purified an alkaline metalloprotease from culture broths of *P. luminescens* and suggested that the protease might be involved in the virulence in *Galleria mellonella* (Lepidoptera, Pyralidae), but there are no supporting data. Proteases trigger a massive programmed cell death of the midgut epithelium in the infected *Manduca sexta* (Lepidoptera, Sphingidae) [18]. Immunocytochemistry studies with an anti-PrtA (protease A) antibody have shown PrtA immunoreactivity associated with the basal lamina of tissues within the insect, suggesting that the protease may attack these membranes that surround individual organs within *M. sexta* [5,18].

PrtA activity can be detectable 24 h after artificial bacterial infection of an insect, suggesting that the protease may play a key role in degrading insect tissues [5]. Some activity might be detectable only with a sensitive and specific substrate, is present in the tissues or in the hemolymph even at 7 to 9 h after infection [16]. The fact that degradation of insect tissues is a determinant in the pathogenic process is also relevant for the symbiosis, because it provides nutrients for the associated nematode, which is unable to grow on insects without a previous bioconversion by the symbiotic bacteria [1]. A study of the expression of PrtA within infected insects supports its suggested role in host bioconversion, as the active enzyme appears in the infected cadaver after the insect has been killed [4]. There are new publications that demonstrate PrtA can play role in the establishment of infection as a pathogenicity/virulence factor for both *Photorhabdus* and *Xenorhabdus* PrtA [14,16,17]. PrtA is a virulence factor with an immune suppression function through the specific cleavage of immune proteins [14].

Here, we find that toxicity varies considerably among insect species and also among strains of the same species. This observation may explain the different results in the previous papers [4–8,11]. Bowen *et al.* [4,5] reported that injected protease is not directly toxic to third-instar *M. sexta* or fifth-instar *G. mellonella* larvae. Our present report reveals that protease purified from broth of *P. luminescens* strain 0805-P5G can be classified as a metalloprotease and showed high toxicity with injection against larvae of *G. mellonella*. Moreover, the protease is also toxic (although less toxic than injection) when administered orally to *P. xylostella* (Lepidoptera, Plutellidae). The insect toxicity on the injection of the protease is surprising on the first sight. Toxicity on injection is not so surprising at the applied quantities (10 μL of 4 ng/μL), because of the estimated high final PrtA concentration in the body of *G. mellonella* (body volume to be 200 μL for *G. mellonella*). But, it gave us the hint that microbiologists and enzymologists might approach using *P. luminescens* protease as a resource for GMO. According to the lack of information about the structure and sequence of each protease, it is difficult to make a realistic comparison of the proteases isolated from different species or even from different strains of the same species. To elucidate the role of the protease in the pathogenesis of insects, molecular cloning and characterization of gene encoding *P. luminescens* strain 0805-P5G protease are in progress in our laboratory.

## 5. Conclusions

In conclusion, a key issue before broad application of such proteases for pest control relates to target specificity. Previous attempts to characterize insecticidal toxicity of protease secreted by *Photorhabdus* had led to considerable confusion in the literature. Our present results demonstrate that the *P. luminescens* strain 0805-P5G protease has a direct toxic effect on two kinds of insects, *G. mellonella* and *P. xylostella*. Although the tobacco hornworm *M. sexta* has been used as a traditional model for insect physiology, it’s not always the best choice. *P. xylostella*, sometimes called cabbage moth, is a possible replacement candidate, for it is one of the most important pests of cruciferous crops in most parts of the world. Larvae can damage leaves, buds, flowers and seed-buds of cultivated cruciferous plants. *P xylostella* was found to have become resistant to biological control by the Bt toxin (*Bacillus thuringiensis*) in the field. This is the first report showing that purified protease of *P. luminescens* is actually toxic to different kinds of insects, including *G. mellonella* and *P. xylostella*. A further investigation is ongoing.

## Figures and Tables

**Table 1:** Toxicities of injected protease against seventh-instar larvae of *G. mellonella.*

Concentration (ng/μL)	Total insects treated^1^	Number of death	Average mortality rate^2^ (%)
11.6	90	90	100
9.5	90	76	84
7.3	90	70	78
6.5	90	65	72
5.8	90	59	66
4.8	90	53	59
3.7	90	43	48

1 The mortality rate for each concentration for 30 insect larvae was recorded after incubation for three days at 25 °C;

2 The average mortality rate was from three experiments.
